# A Comparative Study of Plantar Pressure and Inertial Sensors for Cross-Country Ski Classification Using Deep Learning

**DOI:** 10.3390/s25051500

**Published:** 2025-02-28

**Authors:** Aurora Polo-Rodríguez, Pablo Escobedo, Fernando Martínez-Martí, Noel Marcen-Cinca, Miguel A. Carvajal, Javier Medina-Quero, María Sofía Martínez-García

**Affiliations:** 1 Department of Computer Engineering, Automatics and Robotics, Research Centre for Information and Communications Technologies (CITIC-UGR), University of Granada, 18014 Granada, Spain; auro@ugr.es (A.P.-R.); javiermq@ugr.es (J.M.-Q.); 2 ECsens, Sport and Health University Research Institute (iMUDS), Department of Electronics and Computer Technology, School of Technology and Telecommunications Engineering (ETSIIT), University of Granada, 18014 Granada, Spain; pabloescobedo@ugr.es (P.E.); carvajal@ugr.es (M.A.C.); 3 HCTLab Research Group, Universidad Autónoma de Madrid, 28049 Madrid, Spain; f.martinezmarti@uam.es; 4 Department of Health Sciences, University of San Jorge, Villanueva de Gállego, 50003 Zaragoza, Spain; nmarcen@usj.es

**Keywords:** cross-country ski gear classification, skating instrumented insoles, pressure sensors, IMU, wearable sensors, deep learning

## Abstract

This work presents a comparative study of low cost and low invasiveness sensors (plantar pressure and inertial measurement units) for classifying cross-country skiing techniques. A dataset was created for symmetrical comparative analysis, with data collected from skiers using instrumented insoles that measured plantar pressure, foot angles, and acceleration. A deep learning model based on CNN and LSTM was trained on various sensor combinations, ranging from two specific pressure sensors to a full multisensory array per foot incorporating 4 pressure sensors and an inertial measurement unit with accelerometer, magnetometer, and gyroscope. Results demonstrate an encouraging performance with plantar pressure sensors and classification accuracy closer to inertial sensing. The proposed approach achieves a global average accuracy of 94% to 99% with a minimal sensor setup, highlighting its potential for low-cost and precise technique classification in cross-country skiing and future applications in sports performance analysis.

## 1. Introduction

For several decades, researchers have explored in-shoe plantar pressure systems for applications in health and sports [1]. These systems have been used in diverse contexts, such as human gait analysis [2,3], early ulcer prediction and proactive prevention [4], alpine skiing [5], and cross-country skiing [6,7,8], among others. Pressure detection in these systems relies on various sensing mechanisms, including capacitive, inductive, resistive, piezoelectric, and optical technologies [9]. Among these, piezoresistive sensors have been extensively utilized due to their simplicity and high sensitivity, yielding excellent results in recent studies [10,11,12,13]. Indeed, some commercial in-shoe plantar pressure systems, such as F-Scan^®^ (TekScan, Boston, MA, USA) and ParoTec (Paromed Medizintechnik GmbH, Neubeuern, Germany) [14,15], are based on this technology.

In sports, where movements are rapid, multidimensional data can provide crucial insights for performance analysis. To address these demands, multisensory techniques integrating plantar pressure sensors with inertial measurement units (IMUs) have been proposed. This integration enhances data richness by capturing additional parameters, such as foot angle, angular velocity, and acceleration. Such multisensory approaches have been widely applied in research for various purposes, such as center of pressure trajectory estimation and kinematic gait parameter analysis in walking and running [16], gait analysis during lower limb injury rehabilitation [17], freezing-of-gait detection during walking [18], and turning skill assessment in alpine skiing [19].

In addition, several commercial systems incorporate multiple sensor types. For instance, OpenGo (Moticon, Munich, Germany) integrates 16 plantar pressure sensors and a 6-axis IMU [20] for analyzing human foot dynamics [21]. Similarly, XON snow-1 (Tokyo, Japan) is a commercial monitoring system for snow sports, featuring 9-axis sensors (measuring acceleration, angular velocity, and geomagnetism), dual flex sensors, and four pressure sensors per foot [22].

Despite their usefulness, these instrumentation systems used in health assessment, training, or competition often generate massive amounts of data, which can be challenging to process and analyze. The rapid growth of artificial intelligence (AI) in recent years has facilitated the development of solutions to these challenges in both medical [23,24,25] and sports applications, such as skiing [26,27]. For example, the commercial Carv system, specifically the model Carv 1 [7], designed for alpine skiing, uses machine learning (ML) algorithms in a mobile application to evaluate performance. This system incorporates 72 pressure sensors and a combination of accelerometers, gyroscopes, and magnetometers into instrumented insoles, demonstrating the potential of AI-powered systems for advanced sports analysis.

Advancements in sensor technology and machine learning models have enabled the sophisticated analysis of complex human movements [28], including sports, such as cross-country skiing (XCS). This sport poses significant challenges for analysis due to the demanding environment in which it is performed, rendering traditional or visual methods inadequate. XCS can be divided into two distinct styles: classic and skating. Over the past decade, researchers have employed various types of sensors to study these styles, such as inertial sensors [2,6,29,30,31], pressure insoles [7], and force plates [32]. Both classic and skating techniques rely on specific movement patterns known as gears, which are defined by the coordinated actions of the upper and lower body [33,34]. The selection of a gear depends on factors, such as speed, terrain slope, and environmental conditions.

In the skating style of XCS, four gears have been identified [35,36]. Gear 1 (G1), which is not typically used in competitions, involves a unilateral pole push for each ski push. Gear 2 (G2), in contrast, is commonly employed on steep uphill sections and at lower speeds. In this gear, the skis glide with a wide angle between them, and the skiers perform bilateral but asymmetric pole pushes for every two ski pushes. The asymmetry in G2 results in two variations: G2R, in which the poles are angled to the right; and G2L, in which the poles are tilted to the left. As the slope becomes less steep, skiers often transition to Gear 3 (G3), which is characterized by a symmetric bilateral pole plant every ski push. The final gear, Gear 4 (G4), differs from G3 in that the pole plant occurs with every two ski pushes. During both training and competition, skiers switch between these gears to adapt to varying terrain and speed requirements, making the analysis of their performance critical [34]. However, analyzing the performance in XCS remains a complex task due to the challenging conditions on ski tracks, and wearable systems have proven to be particularly effective for studying these techniques [2,6,31].

From the perspective of instrumentation systems, implementing machine learning models in real-time for systems that use extensive sensor arrays (e.g., in the case of the commercial system Carv 1, 72 pressure sensors and 18 IMU sensors) requires significant hardware resources, including a high-performance processor capable of handling such computational loads. The aim of this study is to classify three skating XCS gears—G2R, G2L, and G3—using simple instrumented insoles, placed in the ski boots, with pressure and inertial sensors. The minimal number of pressure sensors was used to reduce the hardware complexity. By reducing the number of sensors, athletes experience less interference with their movements and greater comfort, leading to a more authentic and competition-like training environment. This approach also reduces computational demands and system costs while maintaining acceptable levels of accuracy. In this work, various sensor combinations were evaluated to determine the optimal configuration according to the accuracy requirements.

### Related Works

XCS gears have been the subject of classification in previous studies. Stöggl et al. achieved an accuracy of 90% using accelerometer data from a smartphone attached to the chest in a skating roller-skiing treadmill test [36]. Sakurai et al. placed IMUs on wrist and roller skis and obtained a general accuracy of 95% when identifying skating gears [37]. Seeberg et al. achieved a 99–100% precision when identifying classic XCS gears, using 7 IMUs placed in different body parts [2]. Rindal et al. developed a system to identify classic XCS gears with 93.9% accuracy using several IMUs in the arms and chest [38]. Johansson et al. obtained a 95% accuracy when identifying skating XCS gears intra-skier, and 78% cross-user using a modified handle ski pole that includes a power meter [30]. Our research group published a model to classify skating XCS gears using two IMUs placed on both skis [6]. The overall accuracy obtained in this previous study was 98%, while cross-user accuracy dropped to 90%. Pressure sensors provide interesting information about the XCS technique; therefore, Pavailler et al. implemented pressure insoles in XCS to study the relative edge time and ski angle [8].

This study further develops an XCS gear classification model, which was initially proposed in [39], using data from three pressure sensors embedded in insoles. In an effort to enhance accuracy, this study augmented the previous design in [39] by integrating another pressure sensor and inertial sensors (IMUs) into the insoles, as well as increasing the number of skiers participating in the study.

## 2. Materials and Methods

### 2.1. Experimental Setup

**Figure 1:**
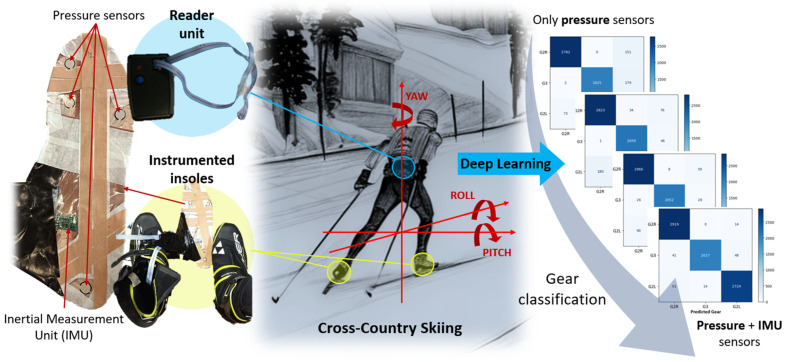
Overview of the developed system based on instrumented insoles with pressure and IMU sensors for cross-country skiing classification using deep learning.

The instrumentation system used in this experiment includes a data logger unit connected to pressure-sensitive insoles and foot movement sensors, which were previously developed by the research group [40,41,42]. The instrumented insoles are equipped with four Flexiforce A201 piezoresistive pressure sensors (Tekscan, South Boston, MA, USA) and an inertial measurement unit (IMU). The IMU comprises a magnetometer, an accelerometer (LSM303DLHC), and a gyroscope (L3GD20), all manufactured by STMicroelectronics (Geneva, Switzerland) (see Figure 1).

The pressure sensors, each with a diameter of 9.53 mm, were strategically positioned at the big toe (BT), the first (1 m) and fifth metatarsal (5 m) heads, and the heel (H). The IMU, located under the arch of the foot, is calibrated to specific settings: a ±8.1 gauss range for the magnetometer, 16 g for the accelerometer, and 2000 degrees per second (dps) for the gyroscope. The datalogger unit is powered by a PIC24FJ256GB106 microcontroller (Microchip Technology Inc., Chandler, AZ, USA), which features multiple I2C, ADC, and SPI ports, among other capabilities. A microSD card reader, interfaced through the SPI ports, stores the insole measurement data in text files at a sampling rate of 100 Hz.

Four experienced skiers participated in this study: Participant A (41 years old, 182 cm, 69 kg), Participant B (44 years old, 180 cm, 82 kg), Participant C (43 years old, 182 cm, 71 kg), and Participant D (47 years old, 180 cm, 85 kg). All participants are highly skilled athletes and coaches with over two decades of experience in cross-country skiing.

Data were collected at the SnoZone indoor ski facility in Arroyomolinos (Madrid, Spain), on a 10% slope within an 18,000 m^2^ area. Each skier performed multiple series of ascents and descents, completing between three and five consecutive runs while using the three targeted skiing techniques: G2R, G2L, and G3. The dataset was structured into five series for training and three series for testing.

To assess the deep learning model’s ability to generalize unseen data and mitigate the risk of overfitting, we employed a 2-fold cross-validation procedure during the evaluation phase. The dataset was collected by the four experts and divided into two equally sized folds. It is crucial to emphasize that during each iteration, data from the four professionals was exclusively assigned to either the training or the testing fold. The performance metric was averaged across both folds to provide a more stable and reliable estimate of the model’s generalization performance, which reduces the influence of any peculiarities or biases that may exist in a single fold.

### 2.2. Sensor Combinations

**Figure 2:**
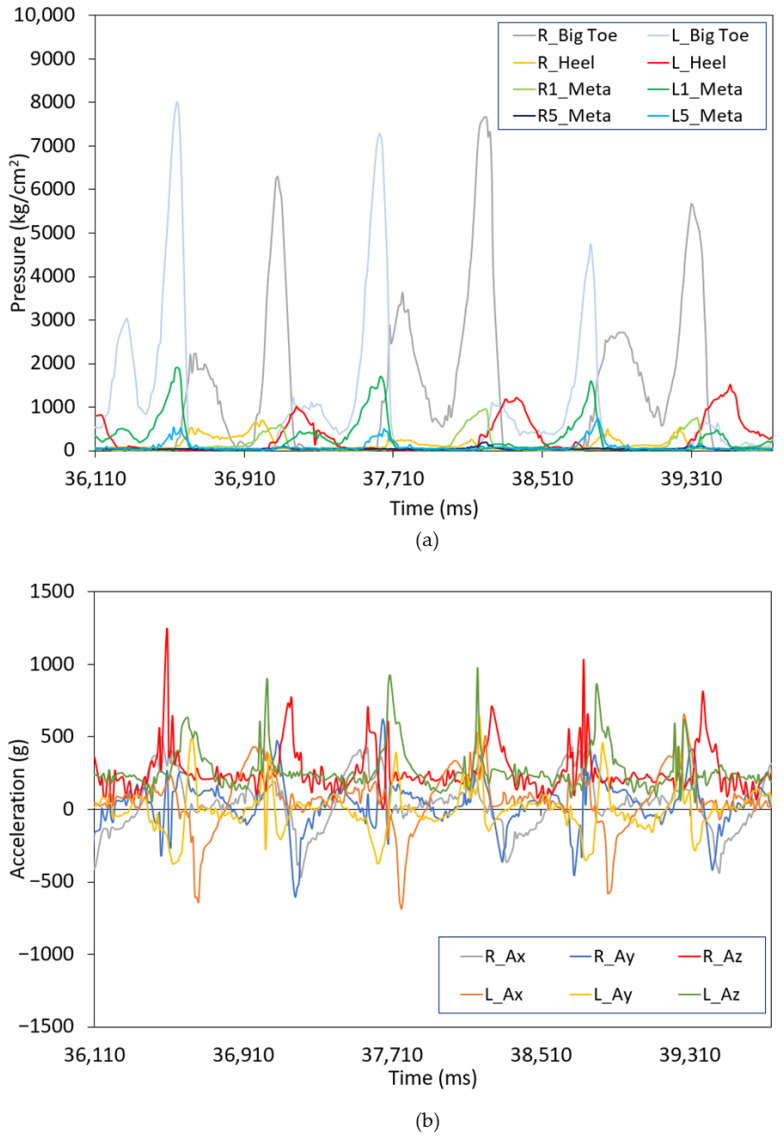
(**a**) Example of the pressure sensor output during the G2R gear for participant A; (**b**) Example of the acceleration (from IMU) during G2R for participant A. In both cases, R refers to the right and L to the left feet.

The instrumented insoles described in the previous section were previously validated for gait [43,44] and jumping activities [40], demonstrating that the distribution and number of sensors are adequate for effectively monitoring these human activities. The pressure sensors provide information during the contact phase with the floor, while the inertial sensors collect data during all phases of movement, as shown in Figure 2. By integrating data from both the pressure sensors and the IMU, the dataset used to train the machine learning model was enhanced. Indeed, this portable system provides a comprehensive analysis of the skiing technique while utilizing a minimal number of sensors.

Figure 2 shows three cycles of G2R. Figure 2a illustrates the pressure flow, while Figure 2b shows the acceleration pattern. G2R is characterized by an asymmetric push of the upper body during the contact phase of the right ski. During the contact phase, the pressure sensors provide values higher than during the non-contact phase (ideally, it should be equal to zero). Thus, the pressure and acceleration patterns can be described as follows: The cycle starts with the left ski contacting and pushing on the snow, while the right ski is in a non-contact phase. The poles are being recovered and prepared to plant, so there is propulsion action from the upper body. The transition of contact from the left to the right ski occurs with a power push and external rotation from the left ski. The right ski contact with the snow and poles is then planted. The poles and right ski are propelled at the same time. Overall, the pressure in both feet follows the same order of pressure flow: first heel, first metatarsals and big toe afterwards, and finally the fifth metatarsals at the end. As it pertains to G2R, it is expected that overall, pressure on the left ski would be greater than that on the right ski. The contact phase of the ski on the snow is similar for both feet: the ski contacts the snow flat, maintaining the smallest possible Z-angle. During the propulsion phase, the ski rotates externally, with the inner edge embedded deeper in the snow.

In the current work, different sets of sensors were evaluated, from the simplest form using two pressure sensors to the inclusion of four pressure sensors and the 9 degrees of freedom of the IMU (the three-dimensional gyroscope, magnetometer, and accelerometer).

A sequential discussion was conducted to analyze the 23 sensor combinations, as follows. Each configuration is labeled as numberP to indicate the number of pressure sensors, with 1 m and 5 m referring to the first and fifth metatarsals, respectively, and *H* for the heel. This notation is followed by the inertial sensors used: *A* for the accelerometer, *G* for the gyroscope, and *M* for the magnetometer.

- No pressure sensors (0P); only one (A), two (AG), or three (AGM) inertial sensors:
  ○ 0P + A: The input to the deep learning model is the output of the 3D accelerometer (A).
  ○ 0P + AG: The outputs of the accelerometer (A) and gyroscope (G) were considered.
  ○ 0P + AGM: All the inertial sensors: accelerometer (A), gyroscope (G), and magnetometer (M), were considered.
- Combinations of two pressure sensors (2P) without inertial sensors:
  ○ 2P.1m5m: Involving the first (1 m) and fifth metatarsals (5 m) sensors.
  ○ 2P.1mH: Considering the first metatarsal (1 m) and heel (H) sensors.
  ○ 2P.5mH: Considering the fifth metatarsal (5 m) and heel (H) sensors.
- Combinations of two pressure sensors on the first and fifth metatarsals (2P.1m5m) with one, two, or three inertial sensors:
  ○ 2P.1m5m + A: Combination of 2P.1m5m plus A.
  ○ 2P.1m5m + AG: Combination 2P.1m5m plus A and G.
  ○ 2P.1m5m + AGM: Combination 2P.1m5m plus A, G, and M.
- Combinations of two pressure sensors on the first metatarsal and heel (2P.1mH) with one, two, or three inertial sensors:
  ○ 2P.1mH + A: Combination of 2P.1mH plus A.
  ○ 2P.1mH + AG: Combination 2P.1mH plus A and G.
  ○ 2P.1mH + AGM: Combination 2P.1mH plus A, G, and M.
- Combinations of two pressure sensors on the fifth metatarsal and the heel (2P.5mH) with one, two, or three inertial sensors (A, AG, or AGM):
  ○ 2P.5mH + A: Combination of 2P.5mH plus A.
  ○ 2P.5mH + AG: Combination 2P.5mH plus A and G.
  ○ 2P.5mH + AGM: Combination 2P.5mH plus A, G, and M.
- Three pressure sensors (3P) without inertial sensors:
  ○ 3P.1m5mH: Involving the first and fifth metatarsals and the heel sensor.
- Three pressure sensors on the first and fifth metatarsals and the heel (3P.1m5mH) with one, two, or three inertial sensors:
  ○ 3P.1m5mH + A: Combination of 3P.1m5mH plus A.
  ○ 3P.1m5mH + AG: Combination of 3P.1m5mH plus A and G.
  ○ 3P.1m5mH + AGM: Combination of 3P.1m5mH plus A, G, and M.
- Four pressure sensors (4P) without inertial sensors:
  ○ 4P: All pressure sensors, involving the first and the fifth metatarsals, heel, and big toe sensors.
- Four pressure sensors (4P) with one, two, or three inertial sensors:
  ○ 4P + A: Combination of 4P plus A.
  ○ 4P + AG: Combination of 4P plus A and G.
  ○ 4P + AGM: Combination of 4P plus A, G, and M.

Based on the observed kinematic and dynamic patterns, the technique performed at each moment can be classified using data from both pressure and inertial sensors. To explore this, the 23 configurations described above were analyzed to evaluate the information provided by the different sensors and their locations to determine their effectiveness in classifying the three techniques with the minimum hardware resources possible.

### 2.3. Data Curation and Segmentation

During data acquisition and cleaning, raw data from the pressure sensors and inertial units were collected and stored in text files, as described in [6,39]. After storage, preprocessing is performed, which involves removing the offset introduced by the pressure sensors and normalizing the data by their area to calculate the pressure in kg/cm^2^. Then, data is cleaned by correcting, removing, or interpolating any missing or corrupted data points. This step ensures data’s integrity and reliability for subsequent analysis. Next, an exponential moving average filter is applied to smooth the data. This reduces noise and fluctuations, revealing the underlying trends and patterns. The temporal smoothing technique further enhances data consistency across time. Finally, a sliding window technique is applied to segment the data into fixed-size windows. The smoothed data is segmented into 5000-ms intervals with 50-ms overlaps. This creates manageable chunks for detailed analysis, allowing us to examine specific time windows and extract meaningful features.

**Figure 3:**
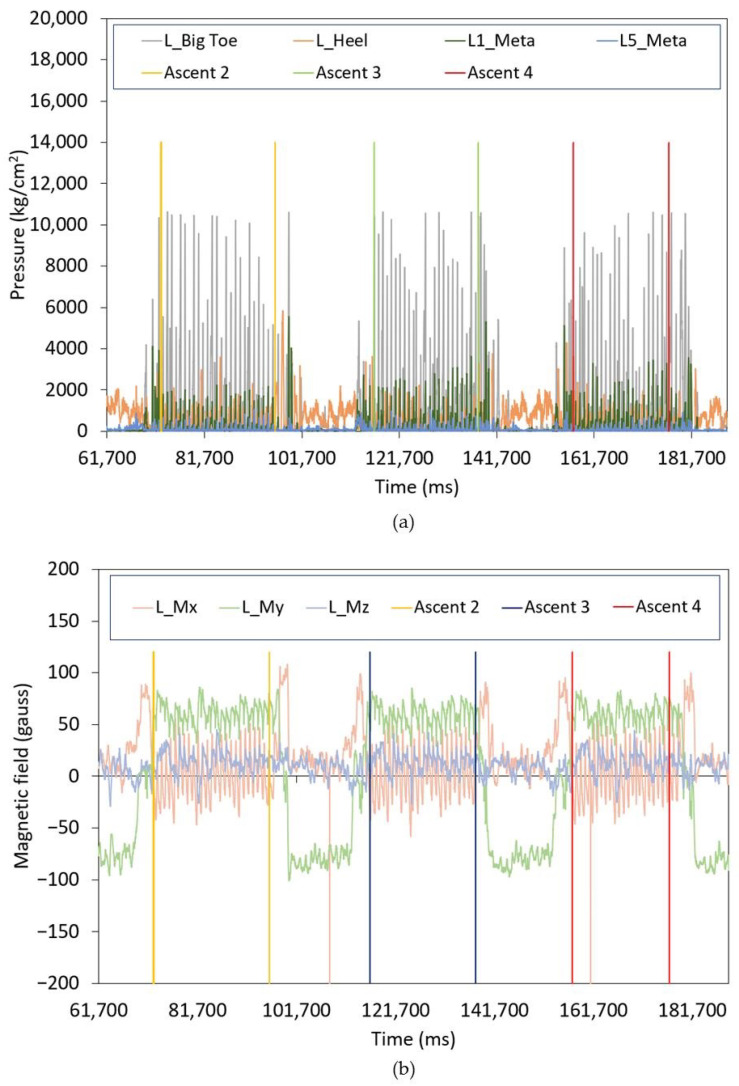
Example of ascent labelling for (**a**) left foot pressure sensors; and (**b**) left foot magnetometer sensor.

Following this, the labeling and feature extraction stages are performed. Each time window is assigned a label based on predefined periods that mark the start and end of various skiing gears, as determined by expert knowledge and linked to the athletes’ ascents (see Figure 3), using the users’ jumps to mark their beginnings and endings. The labeling was also based on the recorded videos of the experiments and the signals obtained from the various sensors in the instrumented insoles. Data from intervening periods, such as the time spent preparing for the next ascent, is excluded from the analysis. Although this exclusion is suitable for controlled testing environments, it may not be applicable in all cases. In future iterations, these transitional periods could be labeled as a separate class, such as ‘preparation’ or ‘transition’, to more comprehensively account for all phases a skier encounters during training or competition.

For each temporal window, the median value is computed for each sensor, serving as the primary statistical feature. The pressure and inertial sensor data are subsequently fused within each time window, creating a consolidated feature set. These fused features, representing each sensor modality, are stored as multidimensional arrays corresponding to the time windows. To ensure consistency in the model’s input scale, the data is normalized for each sensor dimension. This normalization is performed by calculating the mean and standard deviation of the training data, and the same transformation is applied to both the training and validation datasets. This process guarantees that the model receives input features with consistent statistical properties, facilitating improved learning and model generalization. The segmentation and model evaluation were conducted on a computer with an NVIDIA RTX 4070 Ti SUPER GPU (NVIDIA Corporation, Santa Clara, CA, USA), 11th Gen Intel^R^ Core^TM^ i9-11900KF processor (Intel Corporation, Santa Clara, CA, USA) with 3.50 GHz (ASUSTeK Computer Inc., Taipei, Taiwan) and 32 GB RAM (Crucial Technology, Meridian, MI, USA).

### 2.4. Deep Learning Model for Ski-Gear Classification

This study proposes a hybrid deep learning model for skiing gear classification that combines four 1D convolutional layers and two Long Short-Term Memory (LSTM) layers to extract spatial and temporal features from multidimensional sensor data. The input consists of time-series data segmented into fixed-size windows, with feature normalization applied to ensure uniform scaling. The input consists of time-series data, segmented into fixed-size windows, with feature normalization applied to ensure uniform scaling. This time-series data is derived from a set of key features extracted from multidimensional sensor measurements, which capture important patterns for skiing gear classification. The recorded measurements include plantar pressure (expressed in kg/cm^2^, equivalent to 9.8 × 10^5^ Pa), acceleration (expressed in g, where 1 g = 9.8 m/s^2^), angular velocity (in degrees/s, dps), and the magnetic field (in gauss). These measurements were registered along the three Cartesian axes (X, Y, Z) for each foot.

- R1_Meta and L1_Meta: Pressure data from the first metatarsal for the right and left foot, respectively.
- R5_Meta or L5_Meta: Pressure data from the fifth metatarsal for the right and left foot, respectively.
- R_Heel or L_Heel: Pressure data from the heel for the right and left foot, respectively.
- R_Big Toe or L_Big Toe: Pressure data from the big toe for the right and left foot, respectively (measured in kg/cm^2^).
- R_Gx, R_Gy, R_Gz, L_Gx, L_Gy and L_Gz: Gravitational acceleration components along the X, Y, and Z axes for the right and left foot, respectively.
- R_Ax, R_Ay, R_Az, L_Ax, L_Ay and L_Az: Dynamic acceleration components along the X, Y, and Z axes for the right and left foot, respectively.
- R_Mx, R_My, R_Mz, L_Mx, L_My and L_Mz: Acceleration components of the foot in relation to the magnetic field along the X, Y, and Z axes for the right and left foot, respectively, during activity.

The fusion of pressure and inertial sensor data were performed at the feature level, as introduced in Section 2.3. The signals from all sensors were synchronized to ensure alignment. The fusion process consisted of the following steps:

1. Temporal alignment: All signals were resampled to a fixed frequency using linear interpolation.
2. Normalization: The pressure sensor values were normalized using min-max scaling.
3. Feature concatenation: The computed features from both sensor modalities were merged into a single feature vector per time window, which was then input into the deep learning model.

The resulting feature vector includes pressure metrics and inertial metrics, ensuring that the model can learn from both pressure and inertial data in a unified representation, capturing complementary aspects of skiing movements.

The initial stage of the model consists of four one-dimensional convolutional layers (Conv1D) with 16, 32, 64, and 128 filters, respectively, and kernel sizes of 2 and 3. The use of small kernels enables the model to efficiently capture local patterns in the sensor data, which is essential for detecting fine-grained spatial features indicative of specific activities or gear types. The progression from fewer to more filters in successive layers allows for the extraction of increasingly abstract features. The ReLU (Rectified Linear Unit) activation function is employed in these layers due to its ability to introduce non-linearity, enabling the model to learn more complex patterns in the data. To mitigate overfitting, dropout regularization is applied after the second and fourth convolutional layers at a rate of 0.25. This ensures that the model does not overly rely on any specific feature and enhances its generalization ability to unseen data.

The extracted spatial features are then passed into a stacked LSTM layer configuration designed to model temporal dependencies in the sensor data. The first LSTM layer outputs a sequence, allowing the model to maintain a representation of the entire input sequence over time. The second LSTM layer, also with 256 units, aggregates these temporal representations into a single, fixed-length output. This is crucial for capturing long-term dependencies in the data, which is particularly important for tasks that involve recognizing the evolution of a skier’s movements over time. Dropout regularization (0.25) is applied again to prevent overfitting and avoid reliance on any single time step.

The output from the LSTM layers is then processed through a series of fully connected (dense) layers with 2048 and 1024 units, respectively. These layers serve to further refine the learned features, combining them into higher-level representations that enable the model to make the final classification decision. The use of ReLU activation in these layers ensures that non-linear relationships between features are captured. Finally, the model outputs probabilities for the three target classes via a softmax activation function in the final layer. This enables multi-class classification by assigning a probability distribution across the three possible classes for each input sample.

The model was trained using the categorical cross-entropy loss function and optimized with RMSprop. Training was performed over 50 epochs with a batch size of 64, and early stopping based on validation loss was implemented to prevent overfitting [6,39].

The framework for this model is implemented using the Keras library 2.13.1 in Python 3.9.13 (main, 25 August 2022, 23:51:50) [MSC v.1916 64 bit (AMD64)], with TensorFlow 2.13.0 as the backend. This setup allows for efficient training and testing of the model across different sensor configurations. The evaluation of the model is completed using confusion matrices and classification reports, which help to assess the performance of the model on validation data. Data collection corresponds to 44.66 min of recorded data from the four participants (A, B, C, and D) described in Section 2.1.

The proposed model advances upon prior approaches by achieving a weighted average accuracy of up to 99%, surpassing existing state-of-the-art gear classification models that rely on individual sensor modalities [6,30,38,39]. This improvement is largely due to the optimized feature fusion, where the integration of pressure and inertial sensor data enhances classification robustness, outperforming unimodal approaches that use only a single sensor type. An ablation study was also conducted to assess the contribution of each sensor type (see Section 3). The results showed that the combined configuration of four pressure sensors (4P) and an accelerometer–gyroscope module (AGM) achieved superior performance compared to the individual sensor modalities.

## 3. Results

An extensive evaluation of our deep learning model’s performance using data collected from four participants with different sensor combinations was performed to assess its ability to distinguish between different skiing techniques. The different configurations were evaluated to assess the improvement in accuracy with the increase in the number of sensors involved in the deep learning model. This is achieved through the parameter precision per gear and the weighted average accuracy (WAA). In addition, to evaluate the performance of the combinations, the training time and the confusion matrix—used to evaluate the performance of a classification model—were assessed. The matrix provides a detailed breakdown of the model’s predictions compared to the actual gears. WAA and training time were obtained by calculating the average number of epochs per configuration. Although this will be discussed in detail in the following sections, a summary of these two parameters for all sensor combinations is presented in Table 1.

**Figure 4:**
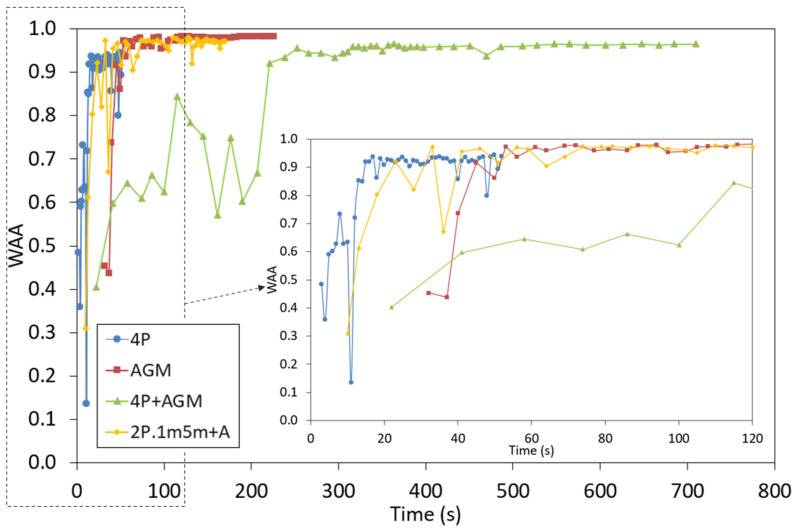
Evolution of the WAA for the configuration with four pressure sensors (4P), with only inertial sensors (AGM), for the joined configuration of both types of sensors (4P + AGM), and finally for the simple configuration 2P.1m5m + A.

Finally, to discuss the contribution of different types of sensors to the learning process, the time required to achieve a WAA higher than 90% was calculated for some selected configurations: 4P, AGM, 4P + AGM, and 2P.1m5m + A, as illustrated in Figure 4. More details will be provided in the following sections.

### 3.1. Classification Using Pressure Sensors

As aforementioned, the average time per epoch and the final WAA achieved are summarized in Table 1. Remarkably, only two pressure sensors achieved a WAA of 94%, which increased up to 95% when data from the fifth metatarsal was included. This highlights the significant contribution of the fifth metatarsal to accurate gear classification. In fact, from the configuration 2P.1mH, the WAA did not increase when the heel and/or big toe sensor was included in the dataset for training the model.

Regarding the time per epoch, the 4P configuration is the slowest (see Table 1). As expected, the training time increases as the number of pressure sensors increases.

The 2P.1m5m and 3P.1m5mH configurations were previously evaluated in two subjects [39], obtaining a WAA of 83% and 92%, respectively. In the current work, the WAA has been improved for both configurations due to the inclusion of two new subjects in the dataset, increasing up to 95% and 94%, respectively.

**Figure 5:**
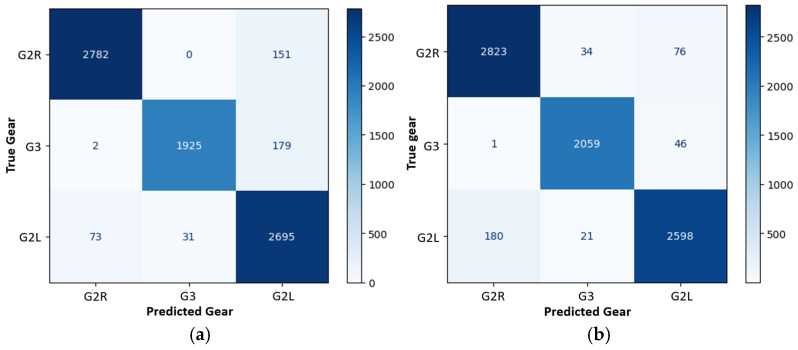
Confusion matrix for gear classification: (**a**) Using two pressure sensors on the first and fifth metatarsals (2P.1m5m); and (**b**) including all four pressure sensors (4P).

On the other hand, when comparing the 2P.1m5m configuration with the 4P configuration, the precision of the G2L gear was higher in all four 4P configurations than in the 2P.1m5m configuration, while the other two gears maintained similar accuracy (see Table 2 and Table 3). Therefore, the use of the 4P combination with additional pressure sensors proves to be more suitable for gear detection, as expected. In fact, the confusion matrix presented in Figure 5 demonstrates high precision and recall values, indicating that the model effectively distinguishes between various skiing techniques. The precision for the “G2R” and “G3” techniques is particularly notable, which suggests that false positives are minimal in these categories.

To evaluate the learning speed, the evolution of the WAA is displayed in Figure 4 for the four-pressure sensor (4P) configuration. The level of WAA (over 90%) was achieved in less than 13 epochs in 15 s, demonstrating the good performance of the deep learning models. In fact, in the first epoch, the WAA was 48%; however, it increased quickly, achieving 72% in the 10th epoch, and 92% in the 13th epoch.

### 3.2. Classification Using Inertial Sensors

**Figure 6:**
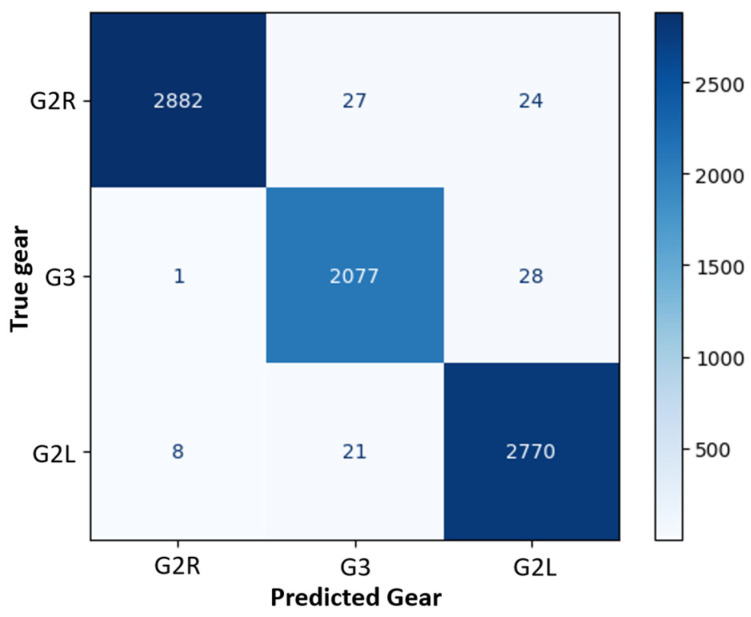
Confusion matrix for gear classification using only inertial sensors (AGM configuration).

During the training phase, using only the inertial sensors in the AGM configuration, there was notable progress, although the initial validation accuracy was lower, at 45% in the first epoch (after 32 s, see Figure 4). Similar to the pressure sensor experiment, the model’s performance improved over time. By the third epoch, the validation accuracy had increased to 74%. By the final epoch, it reached 99%, demonstrating that while the inertial sensors alone were effective, their performance was slightly higher compared to the pressure sensors (see Table 1 and the confusion matrix in Figure 6). Each epoch took an average of 5 s to complete, resulting in a total training time of approximately 230 s, as shown in Figure 4. The confusion matrices revealed that although the model using all the pressure sensors (4P) without inertial sensors performed well (Figure 5b and Table 3), there was a slight decrease in precision and recall compared to the configuration using only inertial sensors (AGM), as seen in Figure 6 and Table 4. Nonetheless, the model achieved high accuracy and F1 scores across all evaluated categories.

The classification using only inertial sensors showed the surprising result that the WAA obtained was the highest among all configurations studied (see Table 1). In fact, the maximum values were obtained when no pressure sensors were considered. This result indicates that the machine learning model obtains very useful information during the fly phase, which was not considered in cases where only pressure sensors were included in the model. The main disadvantage of the inertial sensors is the longer time needed to learn compared to the pressure sensors, as illustrated in Figure 4. This is likely due to the higher complexity of the data reported by the inertial sensors. This increased complexity requires additional resources because of the high computational demands.

### 3.3. Classification with Combined Pressure and Inertial Sensors

**Figure 7:**
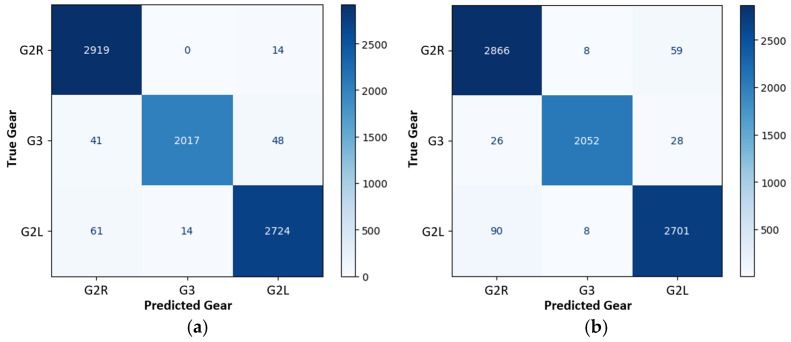
Confusion matrix for gear classification with different combinations of inertial and pressure sensors: (**a**) 4P + AGM configuration; (**b**) 2P.1m5m + A configuration.

The 4P + AGM configuration resulted in a notable performance enhancement. In Figure 4, it can be observed that the validation accuracy started at 40% in the first epoch, but the accuracy steadily increased, reaching 84% by the seventh epoch. In the final epoch, the model achieved an impressive validation accuracy of 98%, reflecting a slight improvement in the performance with respect to the results using only the pressure sensors (95%), as seen in Table 1. Each epoch required an average of 17 s, resulting in a total training time of approximately 850 s (14 min). The confusion matrices (Figure 7a, Table 1 and Table 5) for the combined sensor data demonstrate the model’s superior performance compared to the use of pressure sensors (4P), although slightly lower than the WAA obtained when all inertial sensors (AGM) were used.

To reduce the number of sensors, the performance of the simpler 2P.1m.5m + A configuration was analyzed and compared with that of the more comprehensive combination, as shown in Figure 7b. The WAA obtained was 97% and higher than 96% for the three gears studied (see Table 6), with an average time per epoch of 2 s, which is an important time reduction from the use of only inertial sensors (5 s). In this simplified configuration, if improved accuracy is desired, an increase in the number of users should be implemented.

The evolution of the WAA is displayed in Figure 4, along with the other three main configurations. The configuration that took less time to achieve 90% of WAA was the 4P (15 s), followed by the 2P.1m.5m + A (33 s). The configuration with all sensors (4P + AGM) required more time (221 s) to learn due to the large quantity and complexity of the data. The configuration with only inertial sensors was the combination with a higher final WAA.

Finally, after analyzing the different classifications with various sensor combinations, it can be concluded that the accuracy obtained—whether using only pressure sensors, only inertial units, or a combination of both—ranges from 94% to 99%, as observed in Table 1. The lowest accuracy is found in the combination of 2P and 3P, while the highest is achieved with 0P + AGM.

## 4. Conclusions

The results presented in this study demonstrate the promising performance of both the deep learning model and the sensor configurations, which include pressure and inertial sensors for gear classification in cross-country skiing. The highest average accuracy achieved was 99% using the inertial-only sensor configuration; however, the training time for this setup was approximately 14 min. This time can be significantly reduced by using pressure sensors. For example, the 2P.1m5m + A combination reduced the learning time to less than 3 min (170 s), while still achieving a high final WAA of 98%.

The configuration with the lowest learning time was the one with only the pressure sensors, which only took 50 s to complete and resulted in a WAA of 95%. Therefore, a balance must be considered between training time and WAA. The deep learning model performed exceptionally well across all configurations, with the minimum WAA observed at 94% when using only two pressure sensors. This versatility allows the system to be tailored to meet specific requirements, whether prioritizing maximum accuracy or reducing computational demands and training time.

This wearable system, coupled with the advanced deep learning model presented in this study, provides a powerful and non-invasive solution for studying and classifying gears in cross-country skiing. Its minimal disruption to athletes makes it an ideal tool for performance analysis, technique optimization, and equipment development in cross-country skiing. The flexibility in sensor configurations further enhances its potential for a wide range of applications, contributing to the evolution of athlete training.

## Figures and Tables

**Table 1:** Weighted average accuracy and average time per epoch for all sensor combinations. The average training time data in seconds is rounded to a single significant digit.

					Weighted Avg Accuracy				Avg (t/epoch) (s)			
Pressure Sensors					Inertial Sensors				Inertial Sensors			
	1 m	5 m	H	BT	None	A	AG	AGM	None	A	AG	AGM
**0P**					--	0.97	0.98	0.99		2	3	5
**2P**	×	×			0.95	0.97	0.98	0.98	1	2	6	8
	×		×		0.94	0.96	0.98	0.97	1	2	5	11
		×	×		0.95	0.96	0.98	0.98	1	3	4	11
**3P**	×	×	×		0.94	0.97	0.97	0.97	2	3	7	14
**4P**	×	×	×	×	0.95	0.97	0.97	0.98	3	5	8	17

**Table 2:** Performance evaluation of the model using two pressure sensor data (2P.1m5m).

	Precision	Recall	F1-Score	Support
**G2R**	0.97	0.95	0.96	2933
**G3**	0.98	0.91	0.95	2106
**G2L**	0.89	0.96	0.93	2799
**WAA**	0.95	0.94	0.94	7838

**Table 3:** Performance evaluation of the model using four pressure sensor data.

	Precision	Recall	F1-Score	Support
**G2R**	0.94	0.96	0.95	2933
**G3**	0.97	0.98	0.98	2106
**G2L**	0.96	0.93	0.94	2799
**WAA**	0.95	0.95	0.95	7838

**Table 4:** Performance evaluation of the model using inertial sensor data.

	Precision	Recall	F1-Score	Support
**G2R**	1.00	0.98	0.99	2933
**G3**	0.98	0.99	0.98	2106
**G2L**	0.98	0.99	0.99	2799
**WAA**	0.99	0.99	0.99	7838

**Table 5:** Performance evaluation of the model, including all sensors (4P + AGM).

	Precision	Recall	F1-Score	Support
**G2R**	0.97	1.00	0.98	2933
**G3**	0.99	0.96	0.98	2106
**G2L**	0.98	0.97	0.98	2799
**WAA**	0.98	0.98	0.98	7838

**Table 6:** Performance evaluation of the model for the 2P.1m5m + A configuration.

	Precision	Recall	F1-Score	Support
**G2R**	0.96	0.98	0.97	2933
**G3**	0.99	0.97	0.98	2106
**G2L**	0.97	0.96	0.97	2799
**WAA**	0.97	0.97	0.97	7838

## Data Availability

All data sets and models are available on https://github.com/AuroraPR/Skii-Gears (accessed on 26 January 2025).
